# 636 School Interference and Quality of Life in Pediatric Burn Patients During Acute Recovery

**DOI:** 10.1093/jbcr/iraf019.265

**Published:** 2025-04-01

**Authors:** Abigail Dugan, Carisa Parrish, Sabrina Ung, Casey Lawless, Mallory Netz, Alec Bernstein

**Affiliations:** Children’s Mercy Kansas City Burn Center; Children’s Mercy Kansas City; Children’s Mercy Kansas City; Children’s Mercy Kansas City; Children’s Mercy Kansas City; Children’s Mercy Kansas City

## Abstract

**Introduction:**

Pediatric burn injuries are associated with a range of physical, social, and psychological consequences, including disruption to school adjustment. However, little is known about how smaller burns may interfere with school functioning. The current study evaluates the impact of acute burn injury on school functioning and relationships with demographic and burn-related factors in youth receiving outpatient care in a pediatric burn clinic.

**Methods:**

Data were extracted from retrospective chart review as part of IRB-approved study at a children’s hospital with a pediatric burn center. Participants (N = 102) were school-aged burn patients (M age=9.9 years, 60% female) who attended an outpatient burn clinic < 31 days post-injury and completed the Children’s Dermatology Life Quality Index (CDLQI), which assesses impairments across several quality of life (QOL) domains (e.g., physical discomfort, negative emotions, sleep disturbance, interpersonal problems). The school interference item (scored 0-3) was used to measure potential negative impact on a child’s functioning at school. Spearman’s rho correlations and between-group analyses also included burn total body surface area (TBSA), age, gender, and ethnicity.

**Results:**

Mild to severe levels of school interference were reported by 60% of participants (22.4% mild, 9.2% moderate, 28.6% severe), while 40% reported having no school interference. School interference was correlated with TBSA (ρ =.26, p <.05), age (ρ =.27, p <.05), difficulties coping with burn treatment (ρ =.25, p <.001, and interference with hobbies/playing (ρ =.44, p <.001). School interference was rated similarly by patients with an admission for their burn injury (M =.92, SD =.99) and patients without a hospital admission (M =.98, SD =.88; t(99)=.22, p =.83). Although youth with full thickness burns reported higher levels of total QoL impairments (M = 13.40, SD = 7.02) compared to those with partial thickness burns (M = 8.38, SD = 5.14, t(99)= -2.06, p =.04), there were no group differences on the school interference item in this small sample.

**Conclusions:**

In this small retrospective study, larger burn TBSA and older child age were associated with worse school interference, which in turn was correlated with quality of life impairments (e.g., disruption to playing/hobbies, difficulties coping with burn treatment). This pattern did not vary by ethnic minority status, burn depth severity, or burn-related hospitalization, suggesting that the acute phase of burn injuries can disrupt school for many children.

**Applicability of Research to Practice:**

Understanding the specific effects of burns during childhood is crucial for researchers and practitioners in tailoring interventions and supporting these children during their transition back to daily activities, including school.

**Funding for the Study:**

N/A

